# Abstract of Proceedings of the Buffalo Medical Association

**Published:** 1866-01

**Authors:** Thos. M. Johnson

**Affiliations:** Sec’y.


### ART. III—*Abstract of Proceedings of the Buffalo Medical Association.*

TUESDAY EVENING, Dec. 5th, 1865.

Society met at the usual hour, Dr. Wm. Gould, Vice President, in the chair. Present, Drs. White, Gay, Gould, Samo, Whitney, Greene, Little, Smith, Brown, Rochester and Johnson.

DR. WHITE wished to call the attention of the Association to a case in regard to which an article was published two years since. It will be remembered that a young female, a patient of Dr. Potter, a respectable practitioner of Wyoming county, was, after a protracted labor, delivered with instruments, and that her delivery was followed by severe inflammation of all the pelvic visera, resulting in partial adhesion of the vaginal walls, and complete obliteration of the cavity of the neck of the uterus. It will also be recollected that I then stated that about a year subsequent to her delivery she came to me for treatment, at which time she was nineteen years of age, her general health good, had menstrual molimen and intense pain without any flow.

Recto-vaginal examination showed the uterus in situ, and not above the normal size. On dividing and breaking down the adhesions of the vagina, and dilating it so as to examine the uterus, it was found impossible to discover any entrance to that organ. The neck of the uterus was found to be completely occluded. In the presence of Professors Moore and Eastman, Dr. Coventry and others, a sharp-pointed bistory was carried in the direction of the neck and body of the uterus about an inch and a half without finding any cavity. Sponge tents of increasing size were introduced from time to time. After sufficient dilatation the incision was carried rather more than half an inch farther toward the fundus. Sponge tents were again introduced from time to time for several months, when a galvanic pessary was substituted for the tents and worn for several months. During all this period the patient had severe periodical pains as in dysmonorrhœa, but without menstrual flow. Her general health continued good. At length on one of these occasions a sanguinolent discharge made its appearance. After which pregnancy ensued, and at full term she was delivered of a fine healthy boy, a photograph of which the happy father presented to me soon after.

It will be remembered by the members of the Association that I

have frequently called their attention to the use of the galvanic pessary as a means of stimulus to the uterus. I do not pretend to give the rationale of this case, but the facts are undisputable and full of interest.

Dr. White also related the case of a lady residing in Holland, Erie county, who was brought to him by Dr. Cheesman for diagnosis and treatment. She had consulted several physicians, most of whom had differed in their diagnosis. On examination with a sound Dr. W. found a large post uterine tumor. In order to fully satisfy himself of its character he passed a Simpson's exploring trochar via the vagina into the tumor. A few drops of pus flowed out. There was little or no hemorrhage. The patient was immediately seized wlth pain in the abdomen, looked pale, seemed collapsed. These symptoms of shock were soon followed by marked symptoms of peritonitis. Directed that the patient be kept quiet, and that she have whisky and morphia. Sent for Drs. Rochester and Eastman, with whom he saw the patient an hour later, when she was very feeble, almost pulseless, and death seemed near at hand. On recto-vaginal examination the tumor was found to have completely disappeared. She remained in a critical condition for several days, when she began to improve, and finally entirely recovered her health. What became of the contents of the tumor? They did not pass away by the rectum or the vagina. My belief is that the superior wall of the tumor was very thin and easily ruptured, and that by the introduction of the trochar the superior portion of the tumor *was* ruptured and its contents emptied into the peritoneal cavity, and in due time absorbed. The stimulus and anodynes were continued as long as they seemed indicated, and the patient's recovery was in my belief due to the judicious use of these agents. Dr. W. regards the case an interesting one because of its relation to the question of effusion of pus into the peritoneal cavity and subsequent recovery.

Dr. Gould would inquire of Dr. White how long he thought it advisable for a patient to wear a pessary, and said that he had removed them after they had been carried a year, by direction of some other practitioner.

Dr. White replied that he was not in favor of pessaries. Had no rule as to time. Would use them but a short time without removal. He had not long since removed one that had been introduced by another practitioner, and worn two years, and caused ulceration through the walls of the vagina and rectum.

Dr. Gould reported the case of a female to whom he was called and found all the signs of pregnancy, and thought her labor would come on within a few days. Ten days after called and found the patient quite well, and that the enlargement had entirely passed away.

Dr. Gay mentioned a case of intra-uterine tumor in which three weeks after its removal a tumor was found on the inside of the thigh. On post-mortem examination found that a large amount of pus had passed from the peritoneal cavity down beneath the facia lata to the middle of the thigh and formed the tumor.

Dr. Gould related the case of a woman who, in December, three days after confinement, was taken with chills, tumors soon made their appearance, were opened and succeeded by others until the following May when she recovered.

Report on prevailing diseases being in order, diphtheria, influenza, typhoid and intermittent fevers and diarrhœa were reported as prevailing.

Miscellaneous business being in order the Association unanimously voted that a proper card be placed over the door of these rooms.

The Secretary read a communication from Dr. Joseph A. Peters, tendering his resignation as Secretary of the Association.

On motion of Dr. Rochester the resignation of Dr. Peters was accepted.

The Secretary stated that Dr. Peters had forwarded to him his account with the Association and wished the proper action to be taken in regard to it.

Dr. Rochester moved that it be referred to the auditing committee, to be reported upon at the next regular meeting. Carried. Adjourned. Thos. M. Johnson, Sec'y.

---

Dr. Mott's Will.—The entire value of the estate of Dr. Valentine Mott is stated to be about $400,000. His anatomical museum goes by his will to the New York Medical College.

